# Factor-Based Framework for Multivariate and Multi-step-ahead Forecasting of Large Scale Time Series

**DOI:** 10.3389/fdata.2021.690267

**Published:** 2021-09-10

**Authors:** Jacopo De Stefani, Gianluca Bontempi

**Affiliations:** Machine Learning Group (MLG-ULB), Department of Computer Science, Université Libre de Bruxelles, Brussels, Belgium

**Keywords:** multivariate forecasting, multi-step-ahead forecasting, large scale forecasting, dimensionality reduction, dynamic factor models, nonlinear forecasting, scalability

## Abstract

State-of-the-art multivariate forecasting methods are restricted to low dimensional tasks, linear dependencies and short horizons. The technological advances (notably the Big data revolution) are instead shifting the focus to problems characterized by a large number of variables, non-linear dependencies and long forecasting horizons. In the last few years, the majority of the best performing techniques for multivariate forecasting have been based on deep-learning models. However, such models are characterized by high requirements in terms of data availability and computational resources and suffer from a lack of interpretability. To cope with the limitations of these methods, we propose an extension to the DFML framework, a hybrid forecasting technique inspired by the Dynamic Factor Model (DFM) approach, a successful forecasting methodology in econometrics. This extension improves the capabilities of the DFM approach, by implementing and assessing both linear and non-linear factor estimation techniques as well as model-driven and data-driven factor forecasting techniques. We assess several method integrations within the DFML, and we show that the proposed technique provides competitive results both in terms of forecasting accuracy and computational efficiency on multiple very large-scale (>10^2^ variables and > 10^3^ samples) real forecasting tasks.

## 1 Introduction

The pervasiveness of interconnected devices (IoT) and the consequent big data revolution are shifting the focus of forecasting to problems characterized by very large dimensionality (*n* > 100, … , 1,000), non-linear cross-series dependencies and long forecasting horizons. However, most multivariate forecasting methods in the literature are restricted to low dimension (*n* < 10) vector time series, linear forecasting techniques and short horizons.

The most common approaches to multivariate forecasting are model-driven and data-driven (Januschowski et al., 2020). Model-driven approaches include vector regressions (VAR, VARMA, VARIMA, VARMAX) (Lütkepohl, 2005) and kernel-based regression (Exterkate et al., 2016). Vector AutoRegressive (VAR) models showed a good capability in capturing linear dependencies in applied domains (e.g. wind farm) (Cavalcante et al., 2017). The authors of (Zhao et al., 2018) proposed a correlation constrained and sparsity controlled VAR to reduce the effective number of parameters in model training. However, the main VAR-based model drawback is the parameter size growth at the increase of the lag sample and dimension of the task. Data-driven approaches (notably machine learning) proposed feature-based and representation-based techniques to deal with large-variate settings. Feature-based techniques are based on a multivariate extension of well known univariate forecasting techniques such as *k*-nearest neighbors (Talavera-Llames et al., 2019) or Support Vector (Wang et al., 2020). Such techniques tend to augment the dimensionality of the original data by adding expert-driven combinations of the original features. However, the time required to identify such features and the overhead introduced by the presence of additional features hinders the applicability of such techniques, especially for large dimensions. For this reason, representation based Deep-Learning (DL) methods have been more and more adopted because of their success in modeling non-linear dynamic cross dependencies between variables. In particular, Recurrent (Agarwal et al., 2020; Smyl, 2020; Hewamalage et al., 2021) and Convolutional Neural Networks (Sen et al., 2019) are the most promising models for predicting multivariate time series. Unfortunately, the training process of such models is characterized by a heavy computational load and a need for specialized hardware making them unsuitable for large-scale settings. Moreover, the lack of interpretability of the model and the automatically determined features hinders the extraction of useful information concerning the relevant variables for forecasting.

To cope with the limitations of the existing approaches, we propose an extension of DFML (Bontempi et al., 2017; De Stefani et al., 2018), a hybrid forecasting technique inspired by the Dynamic Factor Model (DFM) approach, a successful forecasting methodology in econometrics (Forni et al., 2005). This paper is an extension and generalization of the original DFML work and addresses three main aspects: factor estimation, factor forecasting and factor recombination. Factor estimation returns a limited number of latent components (factors) from the original series, on which the forecast is performed. Then, the forecasts of the factors are transformed back to the original dimension to obtain the original forecast. The rationale of this approach is to reduce a high-dimensional multivariate problem to a small set of independent univariate problems, thus simplifying the forecasting task. To the best of our knowledge, this paper is the first systematic comparison of data-driven and model-driven strategies for factor estimation and factor forecasting for multivariate multi-step-ahead forecasting in a very large scale setting (>10^2^ variables and > 10^3^ samples).

In particular, the main contributions of this manuscript are:

• A novel modular and extensible framework for multivariate and multi-step-ahead forecasting combining data-driven techniques and model-driven techniques in a Dynamic Factor fashion.
• The assessment of the impact on the DFML accuracy of several methods for factor estimation, including both traditional and Deep Learning based techniques on real data.
• The assessment of the impact on the DFML accuracy of several methods for factor forecasting, including state-of-the-art techniques from both the statistical and machine learning field, on real data.

This work is organized as follows: Section 2 discusses the theoretical framework of time series forecasting. Section 3 introduces the extensions to the DFML framework and its components. Section 4 presents the benchmark setup while Sections 5 and Section 6 summarize and discuss the main experimental results, respectively. Section 7 concludes the paper outlining some future perspectives.

## 2 Materials and Methods

### 2.1 Mathematical Notation

A univariate time series is represented by a vector **y** of size *N* where *N* is the number of samples and *y*_*t*_ is the time series value at time *t* = 1, *…* , *N*. A multivariate time series is a collection of historical observations of *n* variables sharing the same time index, and represented by a matrix *Y*, with *N* rows and *n* columns. The *jth* column of *Y*, denoted with *Y* [*j*], is the univariate time series associated to the *jth* variable, *j* = 1, *…* , *n*. In the following we will denote matrices in upper-case letters (e.g. *Y*) and scalars either in lower-case (e.g. *y*_*t*_) or with the index notation (e.g. *Y*_*t*_ [*j*]) (cf. Table 1).

**TABLE 1:** Notation table.

Notation	Meaning
*n*	Number of variables
*N*	number of observations
*Y*	matrix of observations of size [*N*, *n*]
*Y* [*t*, ⋅]	*n* dimensional vector denoting the time series value at time *t*
*d*	delay
*m*	autoregressive lag
*H*	forecasting horizon

### 2.2 Time Series Forecasting

**FIGURE 1:**
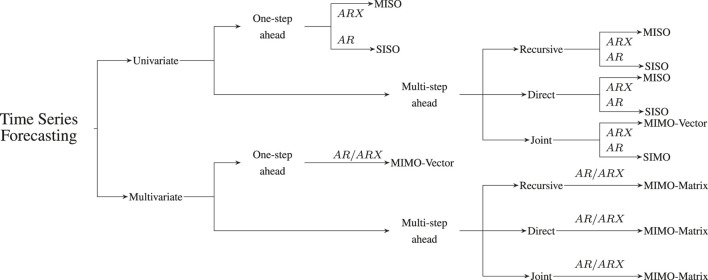
Summary of the different time series forecasting problems and the corresponding tasks. AR indicates an autoregressive hypothesis [i.e. the forecast uses only the information from the past of the considered time serie(s)], while ARX indicates the presence of external regressors.

Time series forecasting deals with the prediction of the future values of a given quantity of interest (a certain time series **y**), given a set of *N* historical observations. A comprehensive overview of the different time series forecasting tasks is presented in Figure 1.

In the univariate one-step-ahead form, this problem is formulated as the estimation of a Single-Input, Single-Output (SISO) auto-regressive mapping $f:R^{m}\mapsto R$

$$y_{t+1}=f\left(y_{t-d},\ldots ,y_{t-d-m+1}\right)+e_{t+1}$$ (1)

where *e* is the noise term, *d* ≥ 0 is the delay and *m* > 0 is called the embedding lag. An embedding procedure represents a time series **y** as a set of input-output pairs (**y** ^(*I*)^, *y*_*t*+1_) with **y** ^(*I*)^ being the *m*-dimensional [*y*_*t*−*d*_, … , *y*_*t*−*d*−*m*+1_] input vector. This formulation is general since it can be employed for estimating both a linear (AR) and a nonlinear mapping (NAR) and enables the adoption of supervised machine learning algorithms (Bontempi et al., 2013). In what follows, for the sake of simplicity, we will assume *d* = 0.

#### 2.2.1 Multi-step-ahead Univariate Forecasting

A multi-step-ahead univariate forecasting consists of predicting the next *H* > 1 values of a time series.

Strategies for predicting univariate time series multi-step ahead have been extensively discussed in (Ben Taieb et al., 2010; Ben Taieb et al., 2012; Bontempi et al., 2013) and can be summarised into two main classes: single output and multiple output strategies.

Instances of the first class are the Iterated and the Direct strategy. The *Iterated* (or Recursive) strategy (Weigend and Gershenfeld, 1994; Cheng et al., 2006; Sorjamaa et al., 2007) learns a one-step-ahead model $f_{REC}:R^{m}\mapsto R$

$$y_{t+1}=f_{REC}\left(y_{t},\ldots ,y_{t-m+1}\right)+e_{t+1}$$ (2)

and then uses it recursively *H* times to return a multi-step-ahead prediction. Though the iterated method is highly sensitive to the estimation error, it has been often used to forecast real-world time series (McNames, 1998; Saad et al., 1998; Bontempi et al., 1999).

The *Direct* strategy (Weigend and Gershenfeld, 1994; Cheng et al., 2006; Sorjamaa et al., 2007) learns independently *H* models $f_{h}:R^{m}\mapsto R$, *h* = 1, *…* , *H*

$$y_{t+h}=f_{h}\left(y_{t},\ldots ,y_{t-m+1}\right)+e_{t+h}$$ (3)

and returns a multi-step-ahead forecast by concatenating the *H* predictions. Since the *Direct* strategy does not use any estimated value as input, it is not prone to the accumulation of one-step-ahead errors. Notwithstanding, no statistical dependencies between the predictions (Kline, 2004; Bontempi, 2008; Bontempi and Taieb, 2011) is considered and these methods often require higher functional complexity (Tong, 1983) than iterated ones in order to model the dependency between two distant instants (Guo et al., 1999).

The *Multi-Input Multi-Output* (MIMO) strategy (Bontempi, 2008; Bontempi and Taieb, 2011) (also known as Joint strategy (Kline, 2004)) avoids the simplistic assumption of conditional independence between future values made by the Direct strategy (Bontempi, 2008; Bontempi and Taieb, 2011) by learning a single multiple-output model

$$\left[y_{t+H},\ldots ,y_{t+1}\right]=F_{J}\left(y_{t},\ldots ,y_{t-m+1}\right)+E$$ (4)

where $F_{J}:R^{m}\mapsto R^{H}$ is a vector-valued function (Micchelli and Pontil, 2005), and *E* is a noise vector whose covariance is not necessarily diagonal (Matías, 2005). The MIMO strategy avoids the conditional independence assumption made by the Direct strategy as well as the accumulation of errors of the Iterated strategy. So far, this strategy has been successfully applied to several real-world multi-step time series forecasting tasks (Bontempi, 2008; Ben Taieb et al., 2009; Ben Taieb et al., 2010; Bontempi and Taieb, 2011).

#### 2.2.2 Multivariate Forecasting

Here we extend the notions of the previous section to multivariate forecasting, taking into account possible cross dependencies among the time series. According to (Januschowski et al., 2020) there are three main approaches to deal with a multivariate forecasting problem: local modeling, global modeling and hybrid modeling.

In local modeling, the multivariate forecasting task is decomposed into a set of *n* SISO or MISO tasks. In the case of SISO tasks, each of the *n* forecasting tasks is treated as an independent problem, thus ignoring the cross dependencies with the other series. In the case of MISO tasks, multiple series can be used as input covariates to forecast a single time series.

$$\left\{\begin{array}{cc} Y_{t+1}\left[1\right] & =f_{1}\left(Y_{t}\left[1\right],\ldots ,Y_{t-m+1}\left[1\right],\ldots ,\right.\\ \; & \left.Y_{t}\left[n\right],\ldots ,Y_{t-m+1}\left[n\right]\right)+e_{t}\left[1\right]\\ \vdots \;\\ Y_{t+1}\left[n\right] & =f_{n}\left(Y_{t}\left[1\right],\ldots ,Y_{t-m+1}\left[1\right],\ldots ,\right.\\ \; & \left.Y_{t}\left[n\right],\ldots ,Y_{t-m+1}\left[n\right]\right)+e_{t}\left[n\right] \end{array}\right.$$ (5)

where $f_{i}:R^{m\times n}\mapsto R$, *i* = 1, *…* , *n*. Although the choice of ignoring cross dependencies (partially, in the case of MISO task or totally, in the case of SISO) might seem disadvantageous at a first glance, it allows to greatly reduce the model complexity, thus reducing the variance of the model and its computational learning time. Moreover, the local models could potentially be trained in parallel, thus improving even more the efficiency of the training. Due to this reduced computational complexity, local models are often used as benchmarks, and potentially outperforming more complex techniques [such as in the M4 Competition (Makridakis et al., 2020a)].

In global modeling, the multivariate problem is tackled as a single MIMO problem, where the model $F:R^{n\times m}\mapsto R^{H\times n}$ takes the embedding vectors of the *n* time series as input and produces the *H*-step ahead forecasts

$$Y_{t+1}=F\left(Y_{t}\right)$$ (6)

Analogously to the univariate case, we may perform the *Iterated* one-step-ahead strategy

$$Y_{t+1}=F_{I}\left(Y_{t},\ldots ,Y_{t-m+1}\right)+E_{t+1}$$ (7)

or the *Direct* *h*-step-ahead strategy:

$$Y_{t+h}=F_{h}\left(Y_{t},\ldots ,Y_{t-m+1}\right)+E_{t+h}$$ (8)

where $F_{I}:R^{n\times m}\mapsto R^{n}$ represents the single *Iterated* model, while $F_{h}:R^{n\times m}\mapsto R^{n}$ indicates the *h*th *Directed* model. Finally, the *MIMO strategy* can be extended to the multivariate case as well:

$$\left[\begin{array}{ccc} Y_{t+H} & \cdots & Y_{t+1} \end{array}\right]=F_{JM}\left(Y_{t},\ldots ,Y_{t-m+1}\right)+E$$ (9)

with $F_{JM}:R^{n\times m}\mapsto R^{H\times n}$ being the model jointly providing *H*-step ahead forecasts for all the *n* time series. This model category allows to properly model the cross-dependencies between the different time series, by increasing the complexity of the functional mappings that have to be estimated (cf. Eqs. 7–9). The number of parameters to be estimated usually grows quadratically (*O* (*n* ^2^)) with respect to the number *n* of input time series, increasing the computational complexity of the estimation process, and limiting their application as the number of time series increases.

In order to exploit the advantages, and limit the drawbacks of both categories, hybrid approaches have been developed, where both the global and the local approach coexist in different forms. For example in hierarchical forecasting models (Athanasopoulos et al., 2017; Taieb et al., 2017; Wickramasuriya et al., 2015), independent local forecasts are first generated and then brought together in a reconciliation process, in order to return coherent global forecasts. Additional approaches adopt kernel-based methods (Hwang et al., 2016) based on the composition of local models, as well as neural models integrating both local components and global components to perform the global forecast (Sen et al., 2019), or where the output of local models is used as input for the global models (Smyl, 2020).

Finally, a well-known hybrid model category is constituted by dynamic factor models (Stock and Watson, 2010), where the global forecasting problem is reduced to a set of local forecasting problem on a reduced number of components, via dimensionality reduction, in a way that the set of components should account for the variability of the original multivariate time series.

### 2.3 Dynamic Factor Models

The Generalized Dynamic Factor Model (DFM) is a technique for multivariate forecasting originating in econometrics (Forni et al., 2005) [for a detailed review see (Stock and Watson, 2010)]. The basic idea of DFM is that a small number of series (the factors) can account for the time behavior of a much larger number of variables. Such factors are latent, i.e., not directly observable and have to be estimated from the original data. Once estimated, they can be can be forecast instead of the original series, reducing the complexity of the multivariate forecasting process. In more formal terms:

$$\begin{array}{ll} Y_{t+1} & =WZ_{t+1}+E_{Y,t+1} \end{array}$$ (10)

$$\begin{array}{ll} Z_{t+1} & =\overset{m-1}{\underset{i=0}{\sum }}\left(A_{t-i}Z_{t-i}\right)+E_{Z,t+1} \end{array}$$ (11)

where *Z*_*t*_ is the vector of unobserved factors of size *q* (*q* < <*n*), *A*_*i*_ are *q* × *q* coefficient matrices, *W* is the matrix (*n* × *q*) of dynamic factor loadings and the disturbances terms *E*_*Y*_, *E*_*Z*_ (also called idiosyncratic disturbances) are assumed to be uncorrelated. The latent factors follow a vector autoregressive time series process and usually do not have a direct interpretation with respect to the original time series. Note that though the seminal work on DFM adopted a frequency domain approach, we will limit to consider here the time domain only.

The practical implementation of DFMs demands to address two main issues: the estimation of the factors (including their number) and the forecasting of their evolution. According to Stock and Watson (2010) there are three main ways to estimate dynamic factors in literature: the first employs parametric estimation (e.g. maximum likelihood), the second makes use of non-parametric methods (e.g. PCA) and the third relies on Bayesian estimation. In this paper, we will restrict to consider non-parametric methods, both linear (PCA, for which consistency was proved (Stock and Watson, 2010)) and non-linear. In this case, the estimation of the number of components typically relies on visual diagnostics (e.g. scree plots) or information criteria. As far as forecasting is concerned, both one-step-ahead and multi-step ahead forecasting based on VAR have been proposed in the econometric literature. Multi-step-ahead typically adopts either iterated or direct linear strategies (Section 2.2.1). For an extended study on the use of DFM and PCA for the forecasting of 149 monthly macroeconomic variables we refer the reader to Stock and Watson (2002).

## 3 The Dynamic Factor Machine Learner Framework

The DFM rationale is that, if a forecaster is able to obtain accurate estimates of factors, then the task of forecasting could be simplified substantially by using the estimated dynamic factors for forecasting, instead of using all *n* original series themselves. Moreover, when *n* > 10^2^ the dimensionality reduction process allows the forecasting process to become tractable with standard statistical tools, often inapplicable for larger dimensions. The final forecasting performance depends mainly on two aspects: the factor estimation algorithm and the accuracy of the factor forecasting.

In (Bontempi et al., 2017; De Stefani et al., 2018) the authors proposed a machine learning extension of the DFM (called DFML—Dynamic Factor Machine Learner) which 1) relies on a linear (PCA) or nonlinear (autoencoder) technique for dimensionality reduction and 2) forecasts each factor independently using a nonlinear model and a univariate multi-step-ahead forecasting strategy by using out-of-sample assessment. Here, we propose a further extension of the DFML framework, in order to include state-of-the-art components in both the factor estimation and the factor forecasting component. The factor estimation component is augmented by including additional non-linear techniques, namely deep feed-forward neural networks and recurrent neural networks encoder-decoder architectures based on LSTM (Hochreiter and Schmidhuber, 1997) and GRU (Cho et al., 2014) units.

The factor forecasting component is improved by the addition of well-known statistical forecasting techniques, such as those employed as benchmarks for the M4 competition (Makridakis et al., 2020a), namely Exponential Smoothing (Holt, 2004), Theta method (Assimakopoulos and Nikolopoulos, 2000), and a statistical ensemble technique of these benchmarks, as well as machine learning based techniques, such as MIMO lazy-learning technique (Bontempi and Taieb, 2011) and gradient boosting based techniques [such as LightGBM (Ke et al., 2017), among the top performers in the M5 competition (Makridakis et al., 2020b].

Overall, many compositions of linear/non-linear factor estimation and linear/non-linear forecasting are implemented and assessed. High variate multi-step forecasting is indeed one of the most challenging tasks in data science and requires an extremely careful management of the bias/variance trade-off by exploring several alternatives in series encoding and forecasting. For instance a non-linear recurrent factor estimation technique could reduce bias (yet increasing variance) in case of nonlinear low noise dynamics while more conventional statistical techniques may be effective in guaranteeing a lower variance (at the cost of a bias increase) in noisy settings with small number of samples.

**FIGURE 2:**
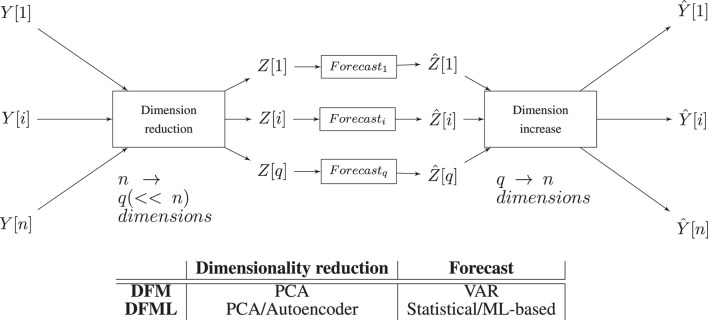
Schema of the DFML architecture with a table summarizing the different components as implemented in the different methods.

The architecture of the DFML, and the comparison with the other models is depicted in Figure 2. It is worth noting that the factor estimation and the factor forecasting modules 1) follow an encoder-decoder like structure (Sutskever et al., 2014) and 2) the two components are decoupled from one another, easily allowing to further extend the architecture by plugging in new components and 3) the complexity of the forecasting step is made independent of *n*.

### 3.1 Factor Estimation

The problem of factor estimation involves the determination of a number of factors *q*, smaller than the original number of time series *n*, such that these factors give a good approximation of the dynamics of the original data. A common approach to produce an estimation of the factor is through dimensionality reduction procedures. A dimensionality reduction procedure assumes that the original multivariate *N* × *n* time series *Y* can be represented in a *q* < *n* dimensional space while retaining as much informative content as possible about the original dynamics. The lower dimension data will then be represented by the *Z* matrix, having dimensions *N* × *q*. Multiple techniques have been developed throughout the years, concerning dimensionality reduction, making both linear assumption about the structure of the lower dimension subspace [e.g. PCA (Hotelling, 1933)], as well as non-linear assumptions [e.g kernel PCA (Schölkopf et al., 1998), autoencoders (DeMers and Cottrell, 1993)] [for a detailed review see Van Der Maaten et al. (2009)]. In this paper we implement three families of dimensionality reduction methods: techniques that do not take into account temporal dependencies, both linear (Section 3.1.1) and non-linear (Section 3.1.2), and techniques that take into account temporal dependencies (Section 3.1.3).

#### 3.1.1 PCA

PCA transforms the *n* original variables *Y* [1], …, *Y* [*n*] into *q* new variables *Z* [1], …, *Z* [*q*] (called *principal components*) such that the new variables are uncorrelated with each other and account for decreasing portions of the variance of the original variables. In other words, PCA performs dimensional reduction by doing orthogonal rotations of the original observed variables. The *q* principal components:

$$Z\left[p\right]=\overset{n}{\underset{j=1}{\sum }}w_{jp}Y\left[j\right],\;p=1,\ldots ,q$$ (12)

are defined as weighted sums of the elements of *Z* with maximal variance, under the constraints that the weights are normalized and the principal components are uncorrelated with each other. It is well-known from basic linear algebra that the solution to the PCA problem is given in terms of the unit-length eigenvectors of the correlation matrix of *Y*. Let us order the eigenvalues *λ*_1_ ≥ *λ*_2_ ≥⋯ ≥ *λ*_*n*_ and the corresponding eigenvector in the matrix *W* of size *n* × *q*. Given the multivariate time series matrix *Y*, *Z* [*p*] = *YW* [, *p*] represents the projection of the series on the *p*th principal component.

Though PCA has been developed originally for independent Gaussian observations, it has been found to be useful also in time series, the most common type of non-independent data. (Jolliffe, 2002). shows that, when PCA is not employed to perform statistical inference (as it is the case of forecasting), non-independence of data should not limit the usage of PCA. The main difference, with non-independent data, is that, while in conventional PCA covariance is computed between variables measured at the same time, in time series it is possible to compute also covariances to model dependencies between variables at different times. (Tsay, 2014). provides an example of application of PCA directly on multivariate time series as well as to the residuals of fitted VAR models, showing its capabilities to find some stable relationships between variables. (Papadimitriou et al., 2005), on the other hand provides an example of summarizing multivariate time series in a streaming setting.

#### 3.1.2 Feed-Forward Autoencoders

Feed-forward autoencoders are a specific category of neural networks trained to learn identity mapping from inputs to outputs (Vincent et al., 2010). Their architecture is characterized by having an input and output layer with the same number of nodes (corresponding to the number of original dimensions *n*) and by the composition of two symmetrical sub-networks: an *encoder*

$$Z_{t}=f_{\theta }\left(Y_{t}\right)$$ (13)

that transforms *n*-dimensional inputs *Y*_*t*_ into some latent (encoded) *q*-dimensional representation *Z*_*t*_, and a *decoder*

$${\hat{Y}}_{t}=g_{\theta^{\prime }}\left(Z_{t}\right)$$ (14)

that reconstructs an *n*-dimensional approximation ${\hat{Y}}_{t}$ of the input *Y*_*t*_ on the basis of the latent *q*-dimensional feature *Z*_*t*_. The two sub-networks are composed solely of feed-forward connections among the layers and might be composed of one or more hidden layers. The networks are usually trained as a single joint network (i.e. the output of the encoder is used as input of the decoder) using gradient descent techniques such as backpropagation, with the objective of minimizing the mean-squared error between the input and the output (Vincent et al., 2010). In their simplest form, the mappings *f*_*θ*_ and *g*_*θ*′_ are linear functions of the inputs and the encoded features *Z*_*t*_ closely related to the PCA principal components (Bourlard and Kamp, 1988). If the hidden layers are non-linear, autoencoders behave very differently from PCA, with the ability to capture multi-modal aspects of the input distribution (Bengio, 2009; Vincent et al., 2010). In this paper we will consider two types of autoencoder, the base version having only one hidden layer in both the encoder and the decoder (henceforth *base*), and a version having two hidden layers in both the networks (called *deep*).

#### 3.1.3 Recurrent Autoencoders

Recurrent Neural Networks (RNN) is a state-of-the-art neural network approach (see (Hewamalage et al., 2021) for a detailed review) where the presence of recurrent connections (i.e. allowing loops in the connection graphs between nodes) allow the modeling of dynamic temporal dependencies. In their simplest form (Graves, 2012), the recurrent connections come from a *hidden state* *H*_*t*_, which is also used for predicting future values *Y*_*t*_:

$$\begin{array}{ccc} H_{t} & = & \sigma \left(W_{HY}Y_{t-1}+W_{HH}H_{t-1}+B_{H}\right) \end{array}$$ (15)

$$\begin{array}{ccc} Y_{t} & = & W_{YH}H_{t}+B_{Y} \end{array}$$ (16)

The matrices *W*_*HY*_, *W*_*HH*_, *W*_*YH*_ represent respectively, the connections between hidden layer *H* and output layer *Y* and the recurrent connections on the hidden layer, while *B*_*H*_ and *B*_*Y*_ indicate the biases of the two layers, respectively. Weights and biases are the learnable parameters of the network, typically by gradient descent algorithms such as backpropagation through time. A nonlinear activation function *σ* (generally a sigmoid function $\sigma_{s}\left(x\right)=\frac{1}{1+e^{-x}}$ or an hyperbolic tangent $\sigma_{t}\left(x\right)=\frac{e^{2x}-1}{e^{2x}+1}$) allows the modeling of nonlinear dependencies, while the recurrent connections allow the modeling of long-term temporal dependencies. For more details concerning backpropagation through time (BPTT) and the internal structure of recurrent cells, we refer the interested reader to Graves (2012). Without loss of generality, the encoder-decoder architecture presented in Section 3.1.2, can be applied with recurrent neural networks:

$$Z_{t}=f_{\theta }\left(W_{ZH}H_{t}+B_{Y}\right)$$ (17)

$$\begin{array}{ccc} {H^{\prime }}_{t} & = & \sigma \left(W_{H^{\prime }Z}Z_{t-1}+W_{H^{\prime }H^{\prime }}{H^{\prime }}_{t-1}+{B\prime }_{H}\right) \end{array}$$ (18)

$$\begin{array}{ccc} {\hat{Y}}_{t} & = & g_{\theta^{\prime }}\left(W_{YH}H_{t}+B_{Y}\right) \end{array}$$ (19)

Where the encoder (Eq. 15 and Eq. 17) and decoder network (Eq. 18 and Eq. 19) will have independent weight and biases matrices. This encoder-decoder architecture is often referred as a sequence-to-sequence (S2S) (Sutskever et al., 2014) model in the literature (Hewamalage et al., 2021). Variations of this architecture (with multiple hidden layers and specific attention mechanisms) have been effectively used in the framework of time series forecasting (Du et al., 2020) and (Bianchi et al., 2017). Additionally, a theoretical study of the sequence-to-sequence framework for time series forecasting, allowing to determine theoretical bounds have been performed by (Kuznetsov and Mariet, 2018). Last but not least, recurrent encoder-decoder architectures have been effectively employed for dimensionality reduction in the signal processing field (Yang et al., 2020), (Susik, 2020).

For these reasons, we assess in this paper two recurrent autoencoders based on LSTM (Hochreiter and Schmidhuber, 1997) and GRU (Cho et al., 2014) units, respectively. The choice is motivated by the fact that in the extensive study (Bianchi et al., 2017) concluded that gated units (such as LSTM and GRU) outperform other recurrent methods when the temporal dependencies can be non-linear and abrupt, and that there is no clear outperformance of LSTM over GRU, or vice versa. Both the LSTM and the GRU autoencoder are implemented as a three layer network: input, hidden and output layers. Both the input and output layer have a number of neurons equal to the number of input time series, a sigmoid activation function and fully connected to the hidden layer. The hidden layer is constituted of a number recurrent cells (LSTM or GRU) equal to the number of factors to estimate.

### 3.2 Factor Forecasting

Once the factors estimated, a forecasting of *Z*_*t*_ is required in order to produce the forecasts for the original series $\hat{Y}$. It should be noted that, though some dimensionality reduction methods (cf. Section 3.1.1) produce decorrelated factors, effectively transforming the original MIMO task into *q* SISO forecasting problems; this does not apply to all the factor estimation methods. For this reason, in this paper, we considered both univariate and multivariate factor forecasting techniques, considering state-of-the-art techniques from both the statistical and the machine learning domain (Januschowski et al., 2020).

#### 3.2.1 Statistical Techniques

Statistical techniques [also called model-driven techniques (Januschowski et al., 2020)] usually define a series of assumptions on the available data, in order to provide a closed-form formulation of the model of the dependency between input and output. We consider here Exponential Smoothing, Theta and Combined method, Single Input - Single Output (SISO) techniques for one-step-ahead forecasting as well as VAR, a Multi Input - Multi Output technique (MIMO) for one-step-ahead forecasting. All those models can be adapted for multi-step-ahead forecasting by implementeing a recursive strategy (Section 2.2). The rationale for considering statistical techniques is that in several forecasting competitions on real-world data (Hyndman, 2020) simple forecasting techniques tend to outperform more complex methods.

##### 3.2.1.1 Exponential Smoothing (ES)

Exponential smoothing is a family (Hyndman and Athanasopoulos, 2018) of SISO forecasting methods, originally introduced in (Holt, 2004), in which the forecasts are computed as a weighted average of the past values and weights decay exponentially with time. In simple exponential smoothing (SES), the forecasts are produced by.

$$\begin{array}{ccc} & {\hat{z}}_{t}=\left(1-\alpha \right)z_{t-1}+\alpha {\hat{z}}_{t-1}=\sum_{i=1}^{t}\alpha {\left(1-\alpha \right)}^{i-1}z_{t-i} & \end{array}$$ (20)

$$\begin{array}{ccc} & 0\leq \alpha \leq 1 & \end{array}$$ (21)

where *z*_*t*_ and $\hat{z_{t}}$ are the value and the forecast of the factor *z*, respectively. The basic method has been extended in to include the historical trend of the time series as well as the presence of seasonality, in both an additive and multiplicative form (Gardner, 1985; Gardner, 2006), leading to the Holt-Winters and the Holt-Winters Damped techniques.

##### 3.2.1.2 Theta

The Theta method (Assimakopoulos and Nikolopoulos, 2000) is based on the combination of multiple one-step ahead SISO individual forecasters, called Theta-lines. Each Theta-line ${y\prime \prime }_{t,\vartheta }$ is constructed by taking a second-order approximation of the original time series, with a specific coefficient *ϑ*. The final forecast is returned by averaging the forecasts produced by the different theta lines.

$$\begin{array}{ccc} & {z\prime \prime }_{t,\vartheta_{i}}=\vartheta_{i}z_{t}^{\prime \prime }=\vartheta_{i}\left(z_{t}-2z_{t-1}+z_{t-2}\right) & \text{with }0\leq \vartheta_{i}\leq 1,t\geq 2 \end{array}$$ (22)

$$\begin{array}{ccc} & {\hat{z}}_{t}=\frac{1}{N_{\vartheta }}\underset{i}{\sum }{z\prime \prime }_{t,\vartheta_{i}} & \end{array}$$ (23)

The Theta ensemble composed by two lines, with the *ϑ*_*i*_ coefficients being respectively equal to 0 and 2, despite its simplicity, outperformed all the competitors in the M3 real-world forecasting competition (Hyndman, 2020) and has been selected as benchmark method for the M4 forecasting competition.

$${\hat{z}}_{t}=\frac{1}{2}\left({z\prime \prime }_{t,0}+{z\prime \prime }_{t,2}\right)$$ (24)

In (Hyndman and Billah, 2003) the author demonstrated the equivalence of the Theta method to a specific form of the exponential smoothing format, in which the drift parameter is half the slope of a linear regression fitted to the data.

##### 3.2.1.3 Combined

Combined (also called Comb) is an ensemble method based on the combination of three SISO one-step-ahead exponential techniques (Gardner, 1985): Single Exponential Smoothing for capturing the level, Holt to linearly extrapolate, and Damped to dampen the linear trend. The combination of the models is obtained by averaging the three outputs

$${\hat{z}}_{t}=\frac{1}{3}\left({\hat{z}}_{t,SES}+{\hat{z}}_{t,Holt}+{\hat{z}}_{t,Damped}\right)$$ (25)

For a detailed description of the methods, we refer the interested reader to the relevant reviews (Gardner, 1985; Gardner, 2006). Unlike Theta, Combined implements an ensemble of heterogeneous forecasting methods, each capturing a different characteristic of the original time series. Like Theta, Combined has been used as benchmark during the different M competitions (Makridakis et al., 2020a).

##### 3.2.1.4 Vector Autoregressive

The *Vector AutoRegressive* model (VAR) is a one-step ahead MIMO model which expresses *Z*_*t*_ as a linear combination of the past values *Z*_*t*−*i*_ of the series with coefficient matrices *A*_*i*_ plus an error vector term *W*_*t*_. The number *m* of considered autoregressive dependencies is also called order of the VAR model. In a compact form the model is:

$$Z_{t}=\overset{m}{\underset{i=1}{\sum }}A_{i}Z_{t-i}+W_{t+1}$$ (26)

where *A*_*t*−*k*+1_, *k* = 1, *…* , *m* is a time-invariant coefficient matrix of size *q* × *q* and *E* [*W* (*j*)] = 0, *j* = 1, *…*, *n*. VAR and state space models have been shown to be equivalent in (Gilbert, 1993). VAR rely on the assumption of stationarity and are typically not suitable for large variate settings (e.g. *n* > 20) because of the high number of parameters to estimate (*mn* ^2^, corresponding to *m A*_*k*_ matrices). For this reasons, we considered the VAR technique as factor forecasting technique inside the DFML, with the number of factors *q* being small, but not as benchmark for the original problem, where the number *n* of variables is too large to make the problem computationally tractable. Like the previous methods, multi-step ahead forecasts are produced from this one-step-ahead method through the *Recursive* strategy.

### 3.3 Machine Learning Based Techniques

Machine learning techniques (or data-driven techniques in (Januschowski et al., 2020)) do not make any parametric assumptions on the data distribution. In this category, we will consider lazy learning (i.e. a single model technique) (Ben Taieb et al., 2012) and gradient boosting (an ensemble technique) (Ke et al., 2017). Those models can be used for multi-step-ahead forecasting both via a *Recursive* and a *Iterative* strategy (cf. Section 2.2). Additionally, we consider a SIMO implementation of the lazy learning model *Joint* (Bontempi and Taieb, 2011), in which all the *h* steps to be forecast are returned by a single model.

#### 3.3.1 Lazy Learning

A lazy learning technique (Aha, 1997) delays the learning phase until the prediction time. In other words, these techniques perform a fit of the model only when a prediction is required by using a computationally efficient technique (e.g. linear). This entails a considerable reduction in the computational cost of the model, while still preserving a good accuracy.

In order to apply local learning tomulti-step time series forecasting, the time series *Z*_*t*_ is embedded into a dataset *D*_*N*_, made up of pairs $\left(X_{\left[t\right]},Y_{\left[t\right]}^{H}\right)$, where *X*_[*t*]_, is a temporal pattern of length *m* including the samples [*Z*_*t*_, …, *Z*_*t*−*m*−1_], and the vector $Y_{\left[t\right]}^{H}$, is the consecutive temporal pattern of length *H*, i.e. the vector [*Z*_*t*+*H*_, …, *Z*_*t*+1_]. In our case, the predictive model is a local weighted regression algorithm (Birattari et al., 1999), where the *h* step ahead prediction of a given sample ${\hat{Y}}_{k}^{h}$ is computed as the average of the *k* most similar samples to the considered sample:

$${\hat{Y}}_{k}^{h}=\frac{1}{k}\overset{k}{\underset{j=1}{\sum }}Y_{\left[j\right]}^{H}$$ (27)

Where $Y_{\left[j\right]}^{H}$ is the output vector of the *j*th closest neighbor. The similarity is defined in terms of a distance metric (e.g. a euclidean distance). The number of neighbors *k* used for the prediction is selected by minimizing the mean squared error with respect to the available values.

In (Bontempi and Taieb, 2011) the authors proposes a MIMO version of lazy learning for forecasting where the number *k* of neighbours is selected through minimization of an estimation of the *h* step forecasting error over a leave-one-out cross-validation procedure. The adoption of a *k*-nearest neighbors lazy learning technique in this study is motivated by two reasons: the reduced computational cost and the capability of the model to exploit local patterns in the data.

#### 3.3.2 Gradient Boosting

Boosting aims to create an accurate forecaster by combining several “weak learners” models [i.e. models characterized by a high bias, and a low variance (Schapire, 1990)]. A boosted ensemble is constructed in a sequential manner, employing a weighting scheme of the samples of the dataset. The first model of the ensemble is defined as a simple average of the available samples $m^{\left[0\right]}\left(z\right)=\frac{1}{N}\sum_{i=1}^{N}z_{i}$ and the weights for all the samples are initialized to the same values. Then, the model is updated via a linear combination, between the learner *l* ^[*j*]^(z) estimated at iteration *j*, and the model constructed at the previous iteration *m* ^[*j*−1]^(z), weighted by the coefficient *ν* ∈ [0, 1]:

$$m^{\left[j\right]}\left(z\right)=m^{\left[j-1\right]}\left(z\right)+\nu l^{\left[j\right]}\left(z\right)$$ (28)

The weights associated with each sample are adapted in a way to increase the weights to those values that have been wrongly predicted. The process is repeated for the desired number of iterations (*J* in this case), and then the final prediction ${\hat{z}}_{t}$ is computed as a weighted sum of the different learners.

$${\hat{z}}_{t}=m^{\left[J\right]}\left(z\right)=m^{\left[0\right]}\left(z\right)+\sum_{j=0}^{j}\nu l^{\left[j\right]}\left(z\right),$$ (29)

The gradient aspect of a gradient boosting method is related to the fact that the sample weight update procedure is performed via a minimization procedure of a given error metric, performed via gradient descent. Moreover, when the chosen error metric is the mean squared error (also called quadratic loss), the gradient boosting procedure is equivalent to training each subsequent model in the ensemble on the residuals of the previous model. For more details concerning the inner workings for the model we refer the interested reader to (Taieb, 2014).

In this paper we include LightGBM (Ke et al., 2017), a gradient-boosted based algorithm specifically optimized to deal with a large number of data instances and a large number of features respectively, implemented with both a *Direct* and *Recursive* strategy.

## 4 Experimental Setup

The experimental study assesses and compares several implementations of the DFML, composing the different factor estimation techniques and factor forecasting techniques discussed in the article. Note, that for the sake of a robust assessment, we set the lag to *m* = 3 and the number of latent factors to *q* = 3 for all the considered methods. In order to improve the readability of the results, we employ the following naming convention: the prefix *DF* is used to indicate a dynamic factor based model, whereas the *UNI* prefix is used to indicate the benchmark, univariate methods used for comparison. In both cases, the prefix is followed by the name of the employed forecasting technique.

### 4.1 Benchmarks

The majority of benchmark techniques used in this article is based on a univariate decomposition of the original *n*-dimensional MIMO task into *n* SISO forecasting tasks. The motivation of this choice is twofold. On one hand, several forecasting competitions based on real data clearly showed the competitiveness of this approach, despite their simplicity (Hyndman, 2020). On the other hand, for several state-of-the-art multivariate techniques a MIMO implementation is either unavailable or computationally intractable due to a large number of variables (e.g. VAR) or the computational cost (e.g. deep learning based methods). Besides the Exponential Smoothing (*UNI-ES*), the Theta (*UNI-Theta*) and the Combined (*UNI-Comb*) we consider the Naive model

$${\hat{Y}}_{t}=Y_{t-1}$$ (30)

a random walk model returning as prediction the latest observation. Despite its trivial nature, in real-world tasks the Naive method is known to outperform more complex learning strategies, especially in presence of continuous sequences of constant values: for that reason it is considered as a baseline to normalize all our accuracy results in Section 4. The methods above are implemented with the code provided for the M4 competition (Center, 2020).

The other multivariate benchmarks are the original Dynamic Factor Model (Forni et al., 2005) (DFM, here DF-PCA-VAR) and the original DFML (Bontempi et al., 2017; De Stefani et al., 2018) (DFML_*PC*_, here DF-PCA-Lazy-DIR and DFML_*A*_, here DF-Base-Lazy-DIR).

### 4.2 Dynamic Factor Machine Learner Framework

We test five different factor estimations techniques and nine different factor forecasting techniques, for a total of 45 different models. The factor estimation techniques are listed below together with the software used for the experiments.

• PCA: the implementation uses the basic R functions cov and eigen.
• base: the base autoencoder is implemented by the rstudio/keras library. The architecture is symmetric with a single hidden layer of size *q* and a ReLU and sigmoid activation functions are used for the hidden and output layer, respectively.
• Deep: the deep autoencoder is implemented by the rstudio/keras library. The architecture is symmetric with three hidden layers [with sizes (10, 5, *q*)], a ReLU activation function for the hidden layer and a sigmoid for the output layer.
• LSTM: The LSTM autoencoder is implemented by the rstudio/keras library. The architecture is symmetric with a single hidden layer (*q* LSTM cells) and a ReLU and a sigmoid activation functions for the hidden and output layer, respectively.
• GRU: The GRU autoencoder is implemented by the rstudio/keras library. The architecture is symmetric with a single hidden layer (*q* GRU cells) and a ReLU and a sigmoid activation function for the hidden and the output layer, respectively.

For all the neural-based techniques, the maximum number of epochs used for the training is set to 50. The factor forecasting techniques are listed below together with the software used for the experiments.

• Comb, ES, Naive, Theta: we use the implementations made available by the M4 competition (Center, 2020). The multi-step-ahead forecast is obtained with a Recursive strategy.
• VAR: the implementation (Eq. 26) is provided by the vars R library and a Recursive strategy returns the multi-step-ahead forecast.
• Lazy-DIR, Lazy-REC, MIMO: these methods denote the lazy learning (Section 3.3.1) with a Direct, Recursive and Joint multi-step-ahead forecasting strategy, respectively. The implementation is made available in the gbonte/gbcode github library by the multisteapAhead function with methods lazydir, lazyiter, mimo respectively.
• LightGBM-DIR and LightGBM-Rec: the implementation is provided by the lightgbm R library. The Direct and Recursive strategies for multi-step-ahead forecasting have been implemented by the authors.

Unless specified otherwise, we employed the default values for the forecasting techniques hyperparameters in the experiments. The entire code used to run the experiments is available in (De Stefani and Bontempi, 2021).

### 4.3 Datasets

We consider three public datasets related to multivariate forecasting problems with high dimensionality (>10^2^ variables and > 10^3^ samples).

• **Electricity consumption** This dataset contains 26,304 samples of 321 variables. Each variable represents the hourly electricity consumption in KWh of 321 clients between 2012 and 2014 (Lai et al., 2018). This dataset has been obtained by preprocessing the original dataset (NREL, 2021) in order to remove null time series and to resample the original data (with a sampling of 15 min) to have an hourly frequency.
• **Traffic usage** This dataset contains 17,544 samples of 862 variables, representing 48 months of hourly data from the California Department of Transportation (Lai et al., 2018). Each variable measures the road occupancy rates (between 0 and 1) returned by sensors monitoring the San Francisco Bay area freeways during 2015–2016 (California Department of Transport, 2021).
• **OBU Mobility data** This dataset contains 1,416 samples of 389 variables. Each variable represents the average hourly occupancy (measured by the number of trucks) of a street in the Brussels region. The original dataset (Bruxelles Mobilité and Machine Learning Group - ULB, 2021) has been preprocessed in order to remove the variables with variance smaller than 0.2, thus reducing the number of variables from 4,529 to 388.

### 4.4 Results Presentation

We consider a rolling window approach (Tashman, 2000) using a window size of *w*_*tr*_ multivariate samples for training, and *H* ∈ {4, 6, 12, 24} multivariate samples for validation. The window size *w*_*tr*_ is set to 2000 samples for the Electricity and Traffic datasets, while for the Mobility dataset, the window size is 400 in order to ensure the feasibility of the rolling approach. It should be noted that this evaluation technique, proposed in (Tashman, 2000) consists of an extension of the well-known cross-validation principle for time-dependent data. All the time series are preprocessed *via* a *z*-score normalization (using the scale R function) and first-order differentiation to de-trend the data. For each window, a multivariate error measure, the Naive Normalized Mean Squared Error (NMMSE) is computed as follows:

$$\begin{array}{ccc} \text{NNMSE} & =\frac{1}{n}\overset{n}{\underset{j=1}{\sum }}\text{NNMSE}\left[j\right] & \end{array}$$ (31)

$$\begin{array}{ccc} \text{NNMSE}\left[j\right] & =\frac{\frac{1}{H}\sum_{h=1}^{H}{\left(Y_{T+h}\left[j\right]-{\hat{Y}}_{T+h}\left[j\right]\right)}^{2}}{\frac{1}{H}\sum_{h=2}^{H}{\left(Y_{T+h}\left[j\right]-Y_{T+h-1}\left[j\right]\right)}^{2}} & \end{array}$$ (32)

where NNMSE averages the univariate NNMSE [*j*] terms.

The statistical significance of the results is assessed *via* a Friedman statistical test (with post-hoc Nemenyi test). For each time series in the multivariate dataset, the considered forecasting techniques are ranked according to their values of NNMSE. Then, the average rank across time series is computed for every forecasting technique and employed as input for the Friedman test (Demšar, 2006). Finally, the post-hoc Nemenyi test is employed to assess the statistical significance of the results of the Friedman test, by determining the value of the critical difference (*CD*). Two forecasting techniques are considered to not have a statistically significant difference if the difference between their average ranks is smaller than the critical difference. In the results visualization, the methods are ordered according to their performance from left to right (the leftmost the best), while the black bar connects methods that are not significantly different (at *p* = 0.05).

We present the results in two formats: 1) a *CD* plot highlighting the statistical significance over all horizons and 2) a tabular format, containing the NNMSE values for different horizons and grouping the methods according to three categories: *DF-Stat* denoting DFM approaches with statistical forecasting, *DF-ML* denoting DFM approaches with machine learning forecasting and *UNI-Stat* denoting univariate statistical baselines.

## 5 Results

### 5.1 Mobility

**FIGURE 3:**
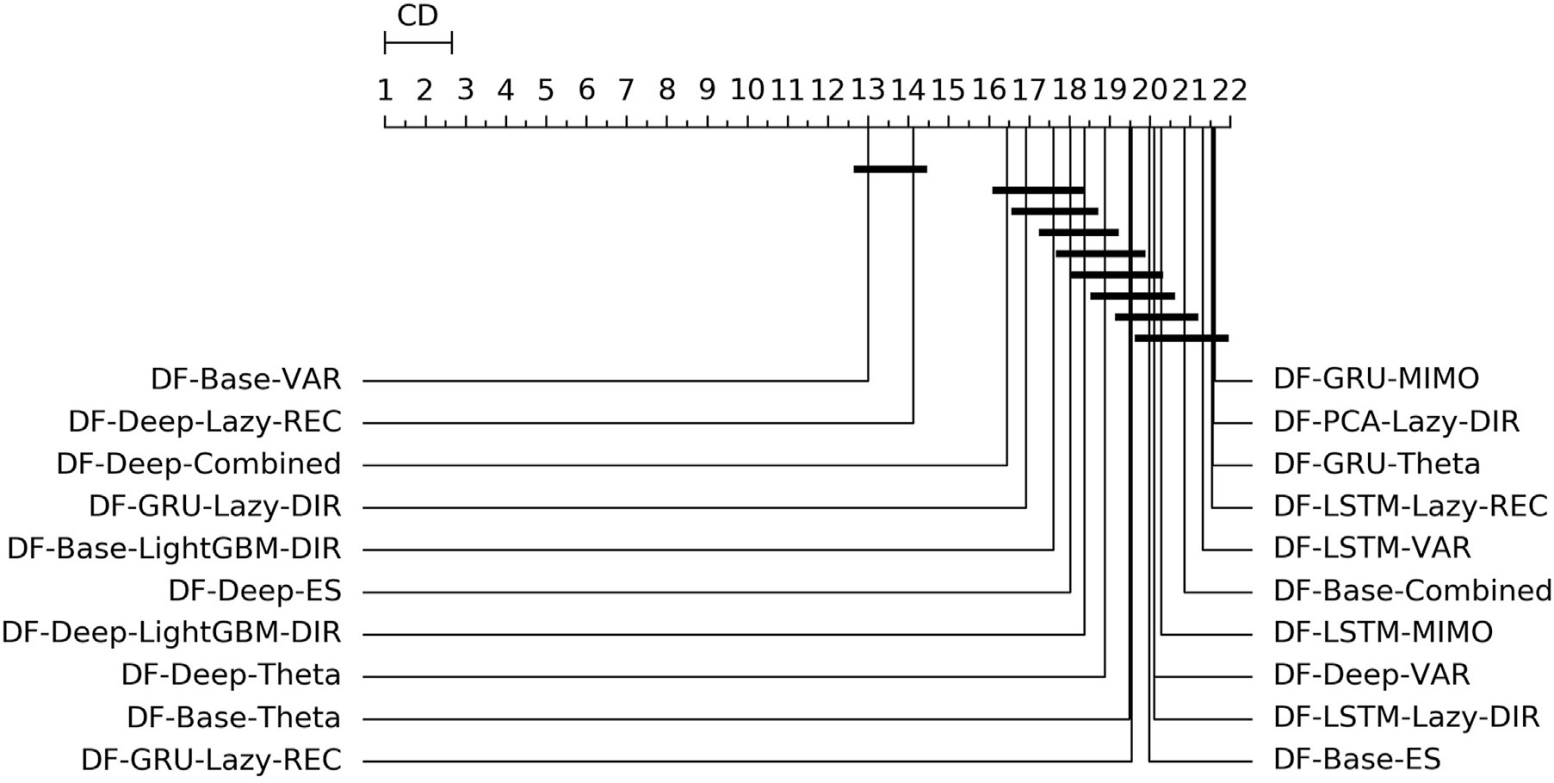
Mobility—Graphical representation according to (Demšar, 2006) of the results of Friedman statistical test (with post-hoc Nemenyi test) comparing the NNMSE of the best 20 methods against each other, aggregated across all horizons *h*. The methods are ordered according to their performance from left to right (the leftmost the best), while the black bar connects methods that are not significantly different (at *p* = 0.05).

Figure 3 shows the overall ranking and the corresponding critical distance according to a Friedman-Nemenyi test (Demšar, 2006). Tables 2, 3 report the average NNMSE for different horizons and groups of methods.

**TABLE 2:** Mobility Naive Normalized MSE for *H* ∈ {4, 6}. The content of the cell *c*_*ij*_ represents the model using the *j*th dimensionality reduction technique with the *i*th forecasting method. The NNMSE for the Naive method is equal to 1. An NNMSE < 1 indicates that the proposed method outperforms the Naive method.

	H = 4					H = 6				
	PCA	LSTM	GRU	Base	Deep	PCA	LSTM	GRU	Base	Deep
DF-Stat										
DF-ES	0.554	0.5477	0.5445	0.5583	0.5475	0.5835	0.5786	0.5767	0.596	0.5764
DF-Theta	0.5541	0.5486	0.5445	0.5582	0.5475	0.5835	0.5789	0.5767	0.596	0.5764
DF-Combined	0.5542	0.5481	0.5448	0.5555	0.5472	0.5837	0.5789	0.5766	0.5961	0.576
DF-VAR	0.5514	0.5448	0.5515	0.5467	0.548	0.5789	0.5777	0.5777	0.5795	0.5768
DF-ML										
DF-Lazy-DIR	0.5435	0.5471	0.5491	0.6037	0.5454	0.5773	0.5783	0.5765	0.6605	0.6161
DF-Lazy-REC	0.5481	0.5479	0.549	0.609	0.5431	0.5793	0.5785	0.5765	0.6868	0.5748
DF-MIMO	0.5546	0.547	0.5514	0.6197	0.5383	0.6001	0.5784	0.5764	0.6422	0.633
DF-LightGBM-DIR	0.5705	0.5558	0.5489	0.543	0.5506	0.6025	0.5813	0.5816	0.5872	0.577
DF-LightGBM-REC	0.5848	0.5465	0.551	0.5855	0.5491	0.6233	0.5834	0.6039	0.617	0.5796
UNI-Stat										
UNI-Naive	1	1	1	1	1	1	1	1	1	1
UNI-ES	0.5494	0.5494	0.5494	0.5494	0.5494	0.5776	0.5776	0.5776	0.5776	0.5776
UNI-Theta	0.5494	0.5494	0.5494	0.5494	0.5494	0.5776	0.5776	0.5776	0.5776	0.5776
UNI-Comb	0.5519	0.5519	0.5519	0.5519	0.5519	0.5794	0.5794	0.5794	0.5794	0.5794

**TABLE 3:** Mobility Naive Normalized MSE for *H* ∈ {12, 24}. The content of the cell *c*_*ij*_ represents the model using the *j*th dimensionality reduction technique with the *i*th forecasting method. The NNMSE for the Naive method is equal to 1. An NNMSE < 1 indicates that the proposed method outperforms the Naive method.

	H = 12					H = 24				
	PCA	LSTM	GRU	Base	Deep	PCA	LSTM	GRU	Base	Deep
DF-Stat										
DF-ES	0.6487	0.6427	0.6336	0.6666	0.6349	0.8048	0.8005	0.8017	0.8007	0.7986
DF-Theta	0.6487	0.6426	0.6336	0.6666	0.635	0.8048	0.8005	0.8017	0.8007	0.7987
DF-Combined	0.649	0.6428	0.6336	0.6685	0.635	0.8048	0.8005	0.8017	0.803	0.7986
DF-VAR	0.6343	0.6359	0.6336	0.6392	0.6334	0.7996	0.7999	0.7992	0.7992	0.799
DF-ML										
DF-Lazy-DIR	0.6282	0.6334	0.6335	0.7084	0.6345	0.7983	0.7998	0.7987	0.8078	0.7994
DF-Lazy-REC	0.6822	0.6335	0.6328	0.7237	0.634	0.8417	0.7999	0.7997	0.8291	0.7983
DF-MIMO	0.6558	0.6339	0.6369	0.6928	0.6431	0.8079	0.7995	0.7997	0.8086	0.8014
DF-LightGBM-DIR	0.6794	0.6399	0.6352	0.6741	0.6348	0.796	0.7992	0.7979	0.7955	0.7957
DF-LightGBM-REC	0.6798	0.6336	0.6341	0.7254	0.6375	0.8335	0.7993	0.7996	0.8296	0.7967
UNI-Stat										
UNI-Naive	1	1	1	1	1	1	1	1	1	1
UNI-ES	0.6334	0.6334	0.6334	0.6334	0.6334	0.7994	0.7994	0.7994	0.7994	0.7994
UNI-Theta	0.6335	0.6335	0.6335	0.6335	0.6335	0.7993	0.7993	0.7993	0.7993	0.7993
UNI-Comb	0.6346	0.6346	0.6346	0.6346	0.6346	0.7997	0.7997	0.7997	0.7997	0.7997

From the analysis of the results we can derive the following considerations:

• Statistical techniques for factor forecasting (*DF-Stat* methods) appear among the top 10 methods (Figure 3).
• Across all the horizons, the non-linear autoencoders (base, Deep, LSTM, GRU) consistently outperform linear factor estimation techniques (PCA). Also, apart from the top two methods, the differences are rarely statistically significant (Figure 3).
• DFML strategies consistently outperform the Naive baseline for different horizons (Tables 2,3).
• The superiority of DFML over UNI-STAT methods (UNI-ES, UNI-Theta, UNI-Comb) is less clear-cut. Taking into considerations all horizons DFML is significantly better than UNI-STAT: nevertheless for large horizons, the accuracy of UNI-STAT and DFML techniques (both DF-Stat and DF-ML) tend to converge.

### 5.2 Electricity

**FIGURE 4:**
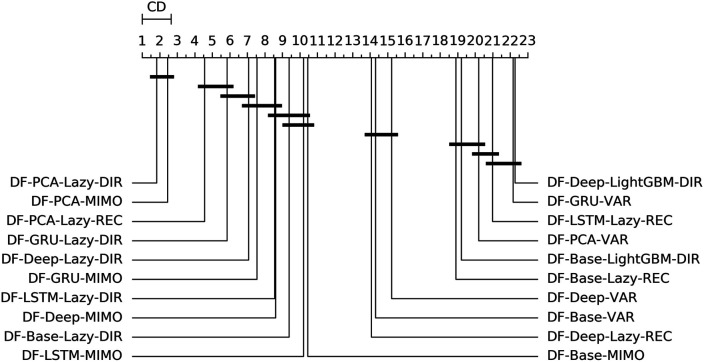
Electricity—Graphical representation according to (Demšar, 2006) of the results of Friedman statistical test (with post-hoc Nemenyi test) comparing the NNMSE of the best 20 methods against each other, aggregated across all horizons *h*. The methods are ordered according to their performance from left to right (the leftmost the best), while the black bar connects methods that are not significantly different (at *p* = 0.05).

Tables 4,5 report the NNMSE, averaged over all the tests sets. Figure 4 shows the ranking and the corresponding critical distance according to a Friedman-Nemenyi test (Demšar, 2006).

**TABLE 4:** Electricity Naive Normalized MSE for *H* ∈ {4, 6}. The content of the cell *c*_*ij*_ represents the model using the *j*th dimensionality reduction technique with the *i*th forecasting method. The NNMSE for the Naive method is equal to 1. An NNMSE < 1 indicates that the proposed method outperforms the Naive method.

	H = 4					H = 6				
	PCA	LSTM	GRU	Base	Deep	PCA	LSTM	GRU	Base	Deep
DF-Stat										
DF-ES	0.5418	0.7396	0.6106	0.4415	0.4424	0.6026	0.6314	0.6594	0.4772	0.4928
DF-Theta	0.5436	0.7482	0.6094	0.4416	0.442	0.5681	0.6306	0.6593	0.4772	0.4884
DF-Combined	0.5402	0.7443	0.6116	0.4391	0.4421	0.6024	0.6325	0.6227	0.4773	0.5236
DF-VAR	0.4684	0.7245	0.4463	0.4149	0.4208	0.4692	0.5191	0.5378	0.4369	0.4326
DF-ML										
DF-Lazy-DIR	0.3202	0.4561	0.3955	0.4318	0.3773	0.3195	0.3746	0.379	0.4018	0.3751
DF-Lazy-REC	0.3297	0.4904	0.4526	0.439	0.3891	0.336	0.4418	0.432	0.4448	0.4684
DF-MIMO	0.3498	0.4331	0.3959	0.4481	0.3716	0.341	0.3869	0.3823	0.4015	0.388
DF-LightGBM-DIR	0.6249	0.5435	0.5902	0.4426	0.4776	0.6985	0.6227	0.6636	0.4936	0.5117
DF-LightGBM-REC	0.56	0.5395	0.5575	0.4895	0.5166	0.6882	0.5866	0.6103	0.5097	0.5567
UNI-Stat										
UNI-Naive	1	1	1	1	1	1	1	1	1	1
UNI-ES	0.5881	0.5881	0.5881	0.5881	0.5881	0.623	0.623	0.623	0.623	0.623
UNI-Theta	0.4722	0.4722	0.4722	0.4722	0.4722	0.499	0.499	0.499	0.499	0.499
UNI-Comb	0.59	0.59	0.59	0.59	0.59	0.6266	0.6266	0.6266	0.6266	0.6266

**TABLE 5:** Electricity Naive Normalized MSE for *H* ∈ {12, 24}. The content of the cell *c*_*ij*_ represents the model using the *j*th dimensionality reduction technique with the *i*th forecasting method. The NNMSE for the Naive method is equal to 1. An NNMSE < 1 indicates that the proposed method outperforms the Naive method.

	H = 12					H = 24				
	PCA	LSTM	GRU	Base	Deep	PCA	LSTM	GRU	Base	Deep
DF-Stat										
DF-ES	0.6357	0.7244	0.6648	0.5075	0.5188	0.6793	2.797	0.6316	0.5959	0.5856
DF-Theta	0.5928	0.7261	0.6648	0.5079	0.5183	0.6388	2.7969	0.6313	0.5957	0.5856
DF-Combined	0.6371	0.747	0.6649	0.5115	0.5575	0.6825	3.3199	0.6334	0.5985	0.6162
DF-VAR	0.4744	0.5188	0.4464	0.4496	0.4649	0.5352	2.2798	0.5358	0.5226	0.5227
DF-ML										
DF-Lazy-DIR	0.2878	0.4179	0.3615	0.4339	0.4621	0.3307	0.4616	0.451	0.485	0.4691
DF-Lazy-REC	0.3747	0.4957	0.569	0.4682	0.4894	0.4429	0.5979	0.5798	0.569	0.5513
DF-MIMO	0.304	0.4445	0.3656	0.4384	0.4878	0.341	0.457	0.4633	0.491	0.4766
DF-LightGBM-DIR	0.7044	0.641	0.5773	0.5217	0.5494	0.3284	0.4988	0.4403	0.4824	0.4683
DF-LightGBM-REC	0.7427	0.5387	0.5251	0.5267	0.5503	0.842	0.5783	0.8388	0.5647	0.5836
UNI-Stat										
UNI-Naive	1	1	1	1	1	1	1	1	1	1
UNI-ES	0.6504	0.6504	0.6504	0.6504	0.6504	0.6746	0.6746	0.6746	0.6746	0.6746
UNI-Theta	0.5295	0.5295	0.5295	0.5295	0.5295	0.5802	0.5802	0.5802	0.5802	0.5802
UNI-Comb	0.6537	0.6537	0.6537	0.6537	0.6537	0.6783	0.6783	0.6783	0.6783	0.6783

On the basis of the results we can make the following considerations:

• Across all the horizons, DFML with non-linear autoencoders (base, Deep, LSTM, GRU) is generally among the best methods (cf. Figure 4). However, some specific factor estimation/forecasting pairs are consistently among the top performers [DF-PCA-{LAZY-DIR,LAZY-REC,MIMO} (Bontempi et al., 2017; De Stefani et al., 2018)].
• Apart from two combinations based on direct gradient boosting, the top 20s only includes lazy techniques (in the top 10s) and VAR (in the bottom 10s) (Figure 4).
• DFML techniques generally outperform the Naive technique and the other univariate benchmarks (UNI-ES, UNI-Theta, UNI-Comb), also for longer horizons. The only exception is represented by the integration of recurrent autoencoders (LSTM, GRU) with statistical techniques (DF-Stat) which performs worse than the UNI-Stat benchmarks (Tables 4 and 5).

### 5.3 Traffic

**FIGURE 5:**
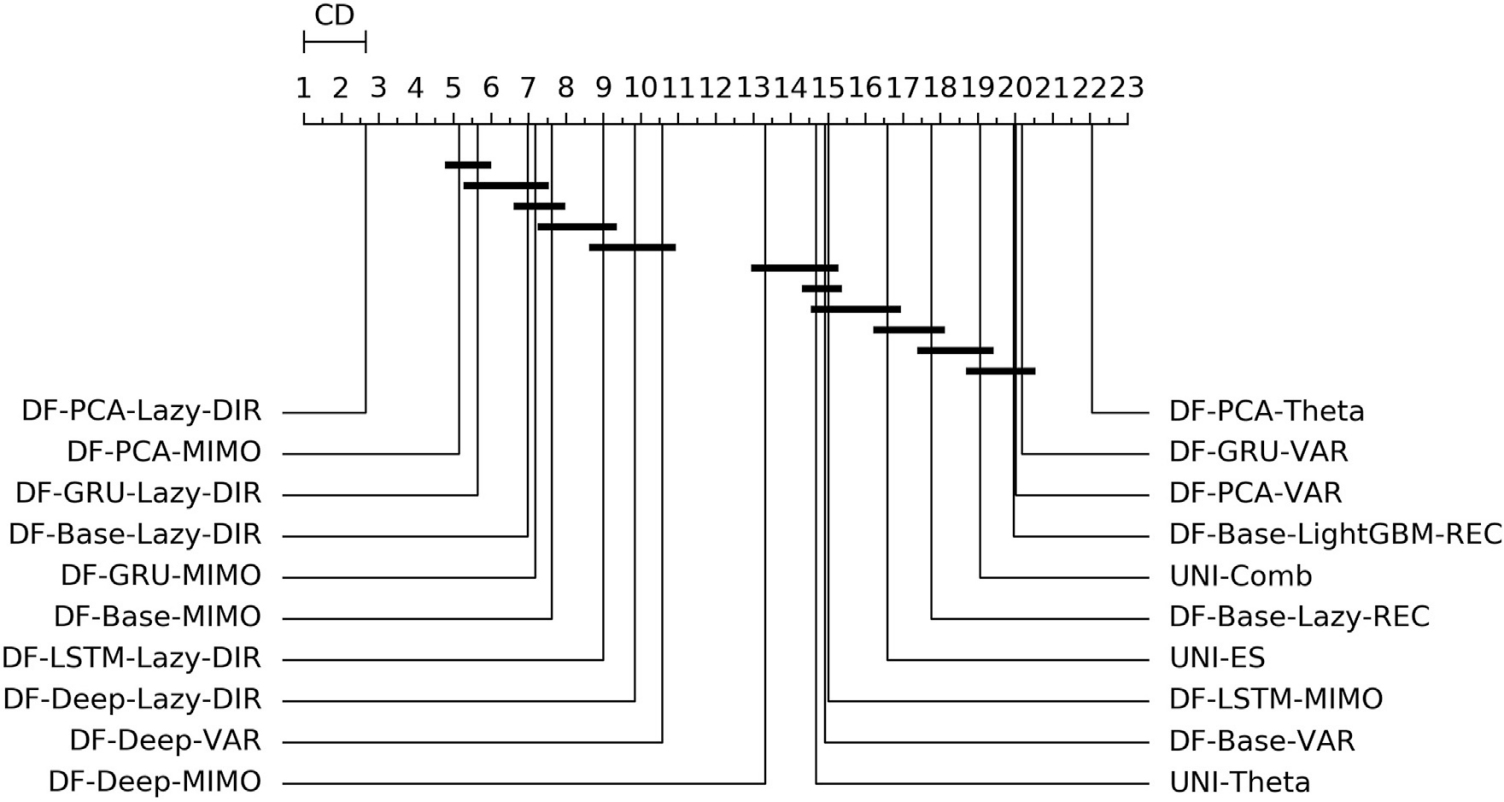
Traffic—Graphical representation according to (Demšar, 2006) of the results of Friedman statistical test (with post-hoc Nemenyi test) comparing the NNMSE of the best 20 methods against each other, aggregated across all horizons *h*. The methods are ordered according to their performance from left to right (the leftmost the best), while the black bar connects methods that are not significantly different (at *p* = 0.05).

Tables 6,7 report the NNMSE, averaged over all the tests sets. Figure 5 shows the ranking and the corresponding critical distance according to a Friedman-Nemenyi test (Demšar, 2006).

**TABLE 6:** Traffic Naive Normalized MSE for *H* ∈ {4, 6}. The content of the cell *c*_*ij*_ represents the model using the *j*th dimensionality reduction technique with the *i*th forecasting method. The NNMSE for the Naive method is equal to 1. An NNMSE < 1 indicates that the proposed method outperforms the Naive method.

	H = 4					H = 6				
	PCA	LSTM	GRU	Base	Deep	PCA	LSTM	GRU	Base	Deep
DF-Stat										
DF-ES	0.5637	0.5819	0.6036	0.4823	0.4788	0.5726	0.4589	0.5386	0.4858	0.4731
DF-Theta	0.5236	0.576	0.6037	0.4823	0.4788	0.5267	0.4583	0.542	0.4857	0.4731
DF-Combined	0.5639	0.5961	0.5731	0.4826	0.5026	0.5729	0.4594	0.5404	0.4835	0.4754
DF-VAR	0.5127	0.6259	0.5115	0.4667	0.4497	0.4718	0.4411	0.4451	0.4538	0.4433
DF-ML										
DF-Lazy-DIR	0.3502	0.437	0.4234	0.4303	0.4659	0.3599	0.3455	0.3719	0.4631	0.3711
DF-Lazy-REC	0.4746	0.399	0.4594	0.445	0.4693	0.4532	0.4775	0.5467	0.6137	0.3735
DF-MIMO	0.3793	0.4264	0.4421	0.436	0.5122	0.3949	0.3378	0.3823	0.4588	0.37
DF-LightGBM-DIR	0.7315	0.6496	0.5325	0.4996	0.6158	0.5644	0.4804	0.4823	0.4648	0.559
DF-LightGBM-REC	0.6973	0.7253	0.6136	0.4998	0.6084	0.6133	0.5077	0.5237	0.5113	0.5183
UNI-Stat										
UNI-Naive	1	1	1	1	1	1	1	1	1	1
UNI-ES	0.5157	0.5157	0.5157	0.5157	0.5157	0.5062	0.5062	0.5062	0.5062	0.5062
UNI-Theta	0.4731	0.4731	0.4731	0.4731	0.4731	0.4645	0.4645	0.4645	0.4645	0.4645
UNI-Comb	0.5226	0.5226	0.5226	0.5226	0.5226	0.5129	0.5129	0.5129	0.5129	0.5129

**TABLE 7:** Traffic Naive Normalized MSE for *H* ∈ {12, 24}. The content of the cell *c*_*ij*_ represents the model using the *j*th dimensionality reduction technique with the *i*th forecasting method. The NNMSE for the Naive method is equal to 1. An NNMSE < 1 indicates that the proposed method outperforms the Naive method.

	H = 12					H = 24				
	PCA	LSTM	GRU	Base	Deep	PCA	LSTM	GRU	Base	Deep
DF-Stat										
DF-ES	0.5786	0.6448	0.583	0.5128	0.5033	0.6083	0.5209	0.5728	0.568	0.5385
DF-Theta	0.5105	0.6089	0.5829	0.5128	0.5037	0.5432	0.5243	0.5728	0.5679	0.539
DF-Combined	0.5792	0.6678	0.583	0.5187	0.5196	0.6097	0.5228	0.5692	0.5881	0.5578
DF-VAR	0.4583	0.4797	0.4958	0.454	0.4453	0.4966	0.5102	0.4993	0.4903	0.4885
DF-ML										
DF-Lazy-DIR	0.3823	0.4649	0.4177	0.4307	0.4325	0.4123	0.4799	0.489	0.4568	0.4557
DF-Lazy-REC	0.4723	0.716	0.5079	0.5039	0.5264	0.6572	41.4895	2.3971	0.5305	0.6124
DF-MIMO	0.4211	0.5064	0.4268	0.4469	0.4401	0.4769	0.5116	0.4933	0.467	0.4529
DF-LightGBM-DIR	0.6798	0.6337	0.5732	0.5711	0.5125	0.4182	0.4606	0.4538	0.4577	0.4698
DF-LightGBM-REC	0.8134	0.5839	0.5478	0.4921	0.4648	0.8062	0.7161	0.7522	0.5216	0.5378
UNI-Stat										
UNI-Naive	1	1	1	1	1	1	1	1	1	1
UNI-ES	0.5154	0.5154	0.5154	0.5154	0.5154	0.5423	0.5423	0.5423	0.5423	0.5423
UNI-Theta	0.4801	0.4801	0.4801	0.4801	0.4801	0.5167	0.5167	0.5167	0.5167	0.5167
UNI-Comb	0.5206	0.5206	0.5206	0.5206	0.5206	0.5446	0.5446	0.5446	0.5446	0.5446

From the analysis of the results we can make the following considerations:

• The main characteristic among the techniques outperforming the univariate benchmarks is the use of a lazy learning technique (either with the Direct (LAZY-DIR) or the Joint (MIMO) strategy) (Figure 5).
• Another recurring forecasting technique in the top 20s is the VAR, in combination with both linear and nonlinear dimensionality reduction techniques (Figure 5).
• DFML strategies consistently outperform the Naive baseline (Table 6 and Table 7).
• The combination of lazy learning with a recursive technique and recurrent autoencoder tends to produce abnormal values, probably due to error propagation or vanishing/exploding gradients problems (Table 7).

### 5.4 Computational Time

**FIGURE 6:**
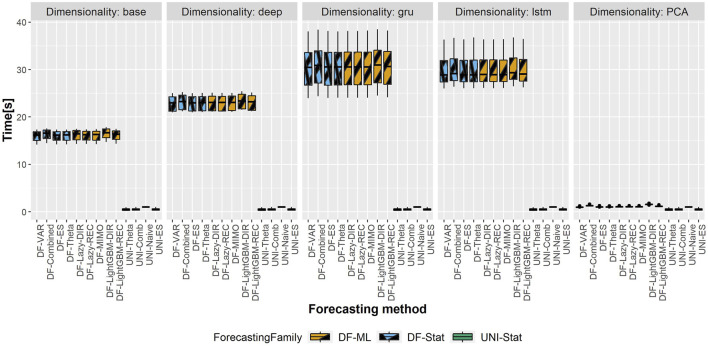
Traffic—Boxplots representing the distribution of the computational time (in s) across the different rolling windows for the shortest horizon (*H* = 4). Each column in the grid represents a different factor estimation technique.

**FIGURE 7:**
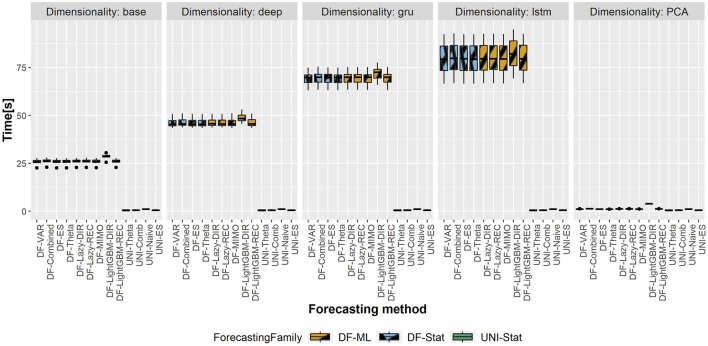
Traffic—Boxplots representing the distribution of the computational time (in s) across the different rolling window for the longest horizon (*H* = 24). Each column in the grid represents a different factor estimation technique.

The total computational time (in seconds) of the different DFML techniques is represented in Figures 6,7, representing respectively, the computational time of the shortest and longest horizon for the dataset having the largest scale (i.e. Traffic). The total computational time includes the time required to train the factor estimation technique and the factor forecasting technique, as well as the time required to generate the forecasts. It should be noted that, while the time required to estimate the factors varies according to the selected techniques, the time allocated to factor forecasting (for a given method) is constant across the different factor estimation techniques, as they employ the same number of components, and therefore the same amount of data. As we can observe in the figure, the majority of the computational time is allocated to the factor estimation technique, with the differences between factor forecasting techniques being negligible (smaller than 1s). The only exception is represented by the LightGBM-DIR technique, where the increase in computational time is justified by the number of models to be trained which is proportional to the forecasting horizon *H*. The fastest technique in terms of computational time is the PCA, while the recurrent based-autoencoder (GRU and LSTM) are the slowest ones. It should be noted that, with the selected number of epochs, the upper bound for all the variants of the DFML is around 75s (Figure 7).

## 6 Discussion

The idea of employing neural components in the framework of a dynamic factor model has already been tested by (Nakagawa et al., 2019) for a MISO one-step-ahead prediction of the returns in the Japanese stock market and by (Kim et al., 2019) in the framework of generative modeling for image reconstruction. However, at the time of writing, to the best of our knowledge, we are not aware of any study implementing neural components in the framework of dynamic factor model for multivariate and multistep ahead forecasting. An additional contribution is constituted by the extensive study, on multiple real datasets, of the different compositions of linear and non-linear factor estimation techniques as well as model-driven and data-driven factor forecasting techniques. We can summarize the findings from our experiments with the following considerations:

• About the *choice of a factor estimation technique* in DFML: linear techniques seem to be the most promising ones, both in terms of forecasting accuracy and computational cost (Figures 4–7). Non-linear techniques both with and without recurrent components are comparable in terms of accuracy: nevertheless, the trade-off between the increase in accuracy and the overhead in terms of computational cost (and the consequent energetic overhead) needs to be carefully taken into account.
• About the *choice of a factor forecasting technique* in DFML: there is no clear winner between model-driven and data-driven techniques. However, two forecasting techniques, VAR (model-driven) and lazy learning (data-driven) appear to be consistently in the top performers across different datasets (Figures 4,5).
• About the *choice of a multi-step-ahead forecasting strategy* in DFML: the Direct (DIR) and Joint (MIMO) strategies consistently outperform the recurrent strategies, confirming the findings of (Bontempi and Taieb, 2011) and (Taieb, 2014) (Figures 4, 5).
• In the majority of the experiments, the DFML is significantly more accurate than the classical DFM (DF-PCA-VAR)), the Naive baseline and the univariate benchmarks (Figures 3,4). Note that outperforming a Naive baseline is not absolutely obvious in multivariate multi-step forecasting as discussed in publications like (Paldino et al., 2021).
• Last but not least, depending on the type of forecasting problem (cf. Section 5.3), univariate factor forecasting techniques still represent a competitive alternative to more complex models (Tables 2,3,6,7).

Further experiments are foreseen to understand the impact of hyperparameters like the number of components *q* or the embedding order of the machine learning models *m*. In addition, the choice of problem specific neural network architecture, fine-tuning of the parameters, as well as longer training times could further improve the performances of the neural-based techniques, if the problem setting allows it.

## 7 Conclusions and Future Work

Multivariate time series forecasting is a major challenge due to the large dimensionality of the available data. In recent years, there has been a remarkable development of multivariate techniques, especially deep learning based ones. The majority of these techniques rely on models of considerable complexity, requiring longer computation times, and often lacking interpretability of the fitted model.

This paper proposes an effective and reliable forecasting methodology based on a combination of model-driven (statistical) and (data machine learning) techniques. The obtained results are promising in terms of scalability and effectiveness. This study supports the idea that factor-based models can be a promising alternative to representation learning strategies, and that the combination of statistical and machine learning techniques (often considered in opposition rather than in synergy) could improve the forecasting performances. Additionally, simpler forecasting methods (e.g., univariate) often neglected, can still provide competitive results (Paldino et al., 2021), even in the case of high dimensional (*n* > 100) multivariate forecasting.

Further studies will focus on an online implementation of the factor-based framework, where both the factor estimation and the factor forecasting component could be incrementally updated as new data samples will be made available, as well as a more scalable implementation of the framework, in order to be able to tackle multivariate series with larger dimensionality. Further attention should be drawn also to the automatic selection of the most relevant parameters characterizing the framework (namely the number of factors for factor estimation and model order for factor forecasting), e.g. extending the preliminary work in De Stefani et al. (2018); Bontempi et al. (2017).

## Data Availability

Publicly available datasets were analyzed in this study. The Electricity and Traffic datasets can be found on the Github page associated to the article (Lai et al., 2018) available at https://github.com/laiguokun/multivariate-time-series-data. The Mobility data analyzed for the study can be found on the corresponding Kaggle page (https://www.kaggle.com/giobbu/belgium-obu) and in (De Stefani and Bontempi, 2021). The original contributions presented in the study are included in the article/Supplementary Material, further inquiries can be directed to the corresponding author.
