# Supplementary figures and images for: Factor-Based Framework for Multivariate and Multi-step-ahead Forecasting of Large Scale Time Series
Source: Front Big Data. 2021 Sep 10;4:690267. doi: 10.3389/fdata.2021.690267 (PMC8460934; doi:10.3389/fdata.2021.690267)

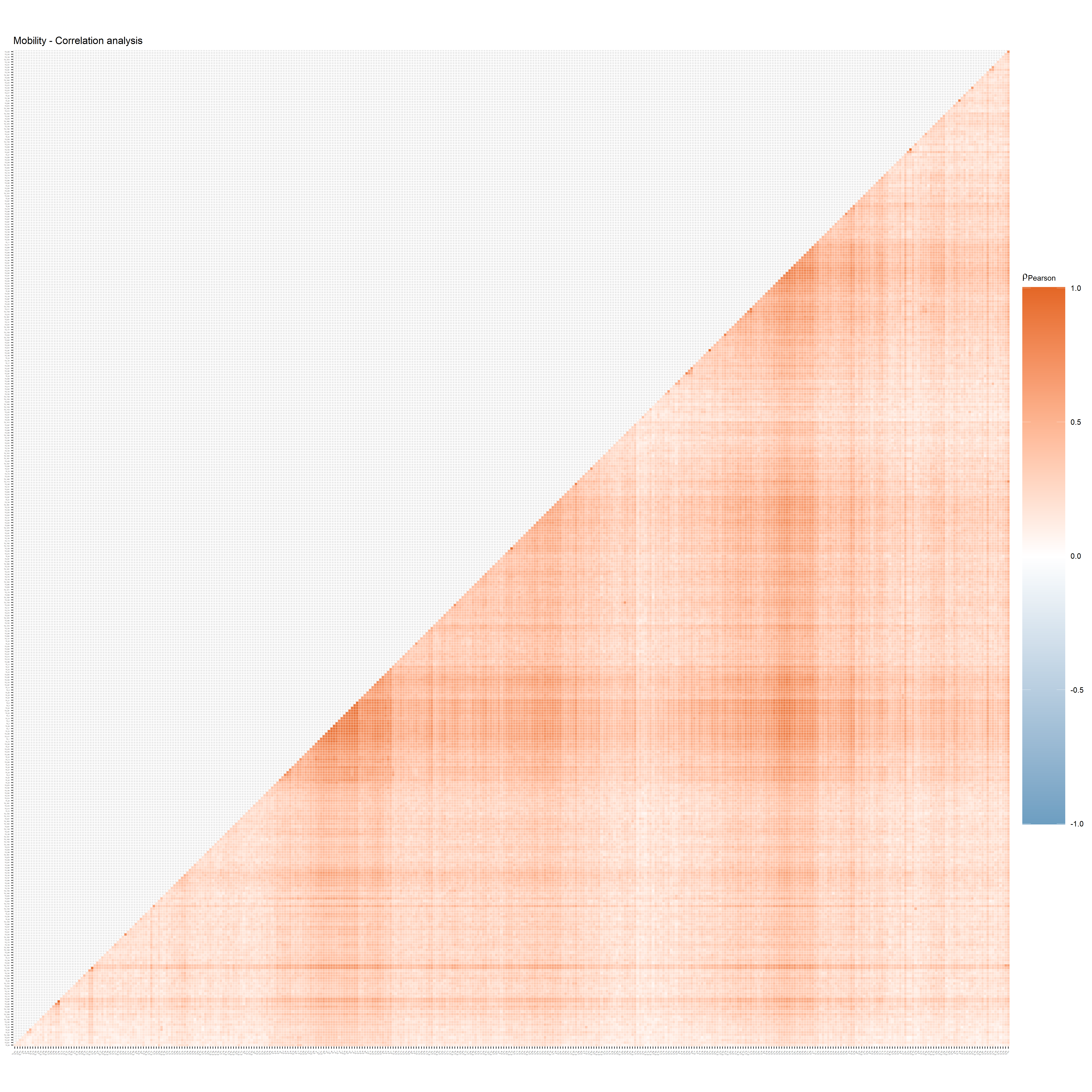

Supplement: Supplementary file 1 [file Image2.PNG]

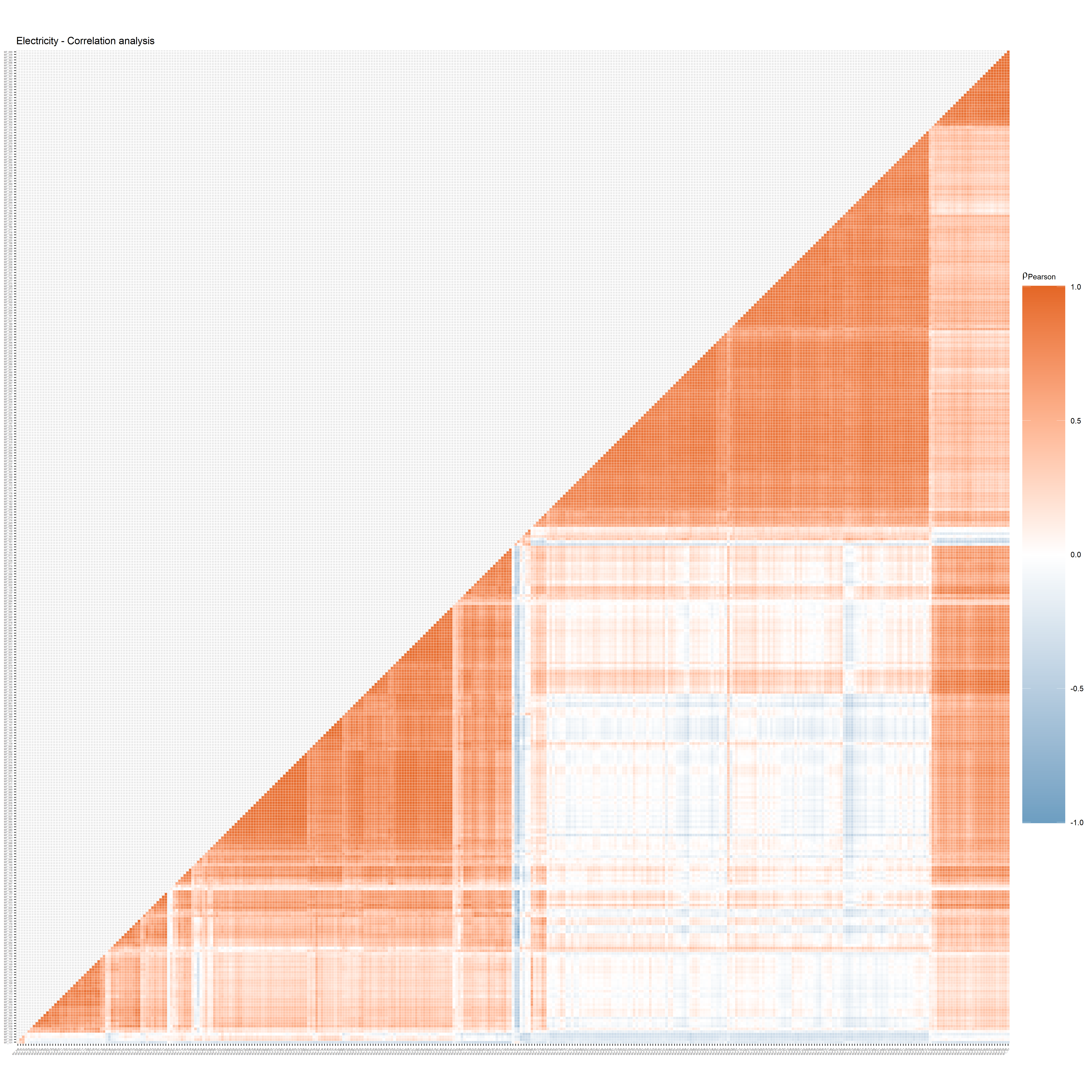

Supplement: Supplementary file 2 [file Image1.PNG]

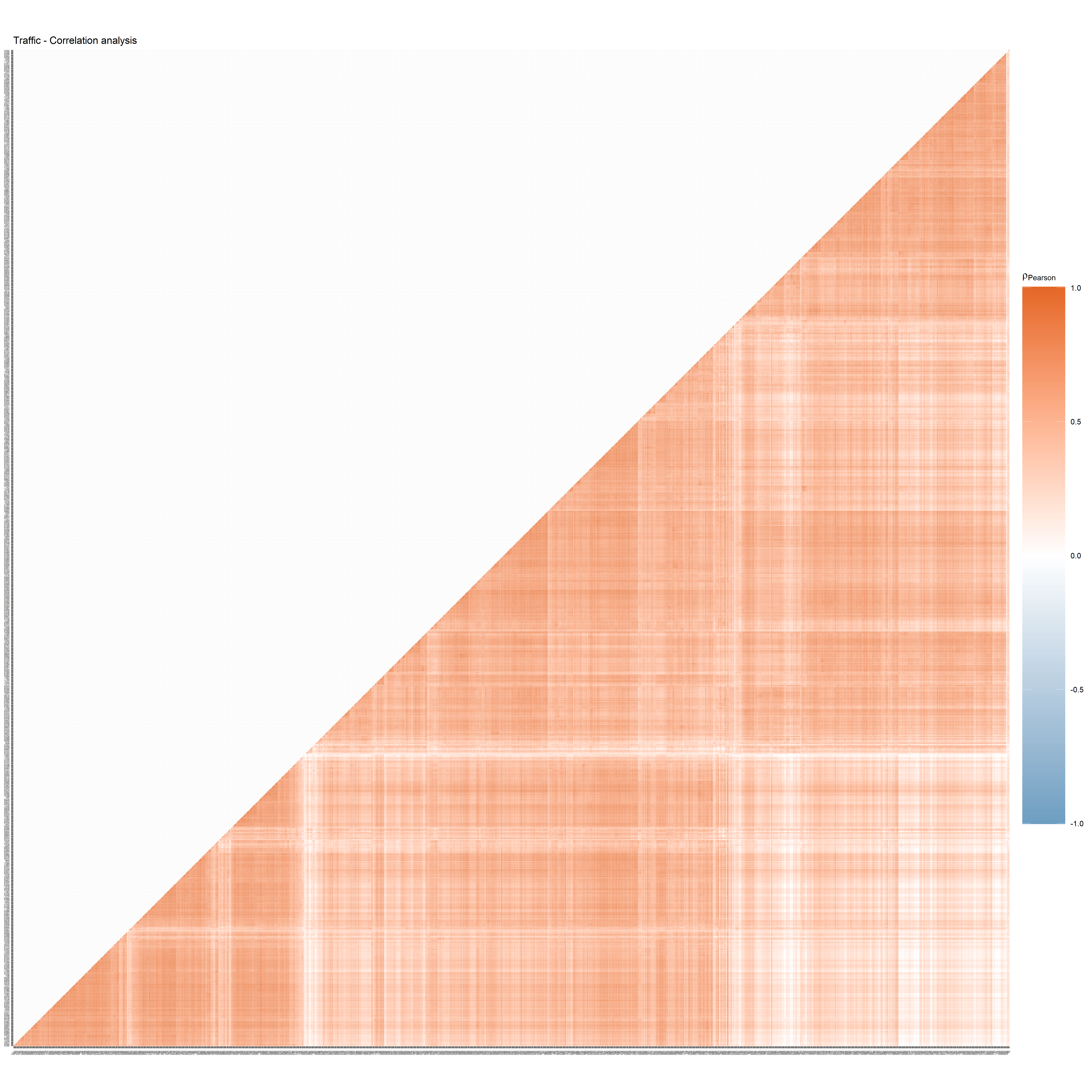

Supplement: Supplementary file 3 [file Image3.PNG]
